# VHL mosaicism: the added value of multi-tissue analysis

**DOI:** 10.1038/s41525-022-00291-3

**Published:** 2022-03-18

**Authors:** Leslie E. Oldfield, Jessica Grzybowski, Sylvie Grenier, Elizabeth Chao, Gregory S. Downs, Kirsten M. Farncombe, Tracy L. Stockley, Ozgur Mete, Raymond H. Kim

**Affiliations:** 1 Princess Margaret Cancer Centre, Toronto, ON Canada; 2 Ambry Genetics, Aliso Viejo, CA USA; 3 Division of Clinical Laboratory Genetics, Laboratory Medicine Program, University Health Network, Toronto, ON Canada; 4 Toronto General Hospital Research Institute, University Health Network, Toronto, ON Canada; 5 Department of Laboratory Medicine and Pathobiology, University of Toronto, Toronto, ON Canada; 6 Princess Margaret Cancer Centre, University Health Network, Sinai Health System, Hospital for Sick Children, Department of Medicine, University of Toronto, Toronto, ON Canada

**Keywords:** Cancer screening, Cancer genetics, Genetic testing, Medical genetics

## Abstract

Von Hippel-Lindau disease (VHL) is an autosomal dominant, inherited syndrome with variants in the *VHL* gene causing predisposition to multi-organ benign and malignant neoplasms. A germline *VHL* variant is identified in 95–100% of individuals with a clinical diagnosis of VHL. Here, we present the case of an individual with a clinical diagnosis of VHL disease where peripheral blood DNA analysis did not detect a *VHL* variant. Sequencing of four tumor tissues (ccRCC, pheochromocytoma, lung via sputum, liver) revealed a *VHL* c.593 T > C (p.Leu198Pro) variant at varying allele fractions (range: 10–55%) in all tissues. Re-examination of the peripheral blood sequencing data identified this variant at 6% allele fraction. Tumor analysis revealed characteristic cytomorphological, immunohistochemical reactivity for alpha-inhibin, and CAIX, and reduced pVHL reactivity supported VHL-related pseudohypoxia. This report of a rare case of *VHL* mosaicism highlights the value of tissue testing in *VHL* variant negative cases.

## Introduction

Von Hippel-Lindau disease (VHL) is an autosomal dominant syndrome that predisposes individuals to benign and malignant neoplasms in various organs^1^. The most common neoplasms affecting individuals with VHL are hemangioblastoma of the retina, hemangioblastoma of the central nervous system (CNS), endolymphatic sac tumor, clear cell renal cell carcinoma (ccRCC), pheochromocytoma (PCC), paraganglioma, and pancreatic neuroendocrine neoplasm or cyst^2^. VHL is caused by a germline pathogenic variant in the tumor suppressor gene *VHL* and has a prevalence of ~1 in 36,000^3^. Tumorigenesis typically follows the two-hit mechanism where the wild-type *VHL* allele becomes lost or inactivated through multiple mechanisms, including somatic methylation of the *VHL* promoter region, point mutations, small insertions/deletions, or loss-of-heterozygosity (often through large deletions)^4^.

Patients with suspected VHL disease undergo confirmation through molecular genetic analysis of the unaffected DNA. A germline *VHL* variant (sequence or copy number variant) is identified in 95–100% of individuals who fulfill the clinical criteria for VHL^5–8^. A study of 945 VHL families found the majority of individuals have a missense variant (52%), followed by frameshift (13%), large deletion (11%), nonsense (11%), splice site (7%), and in-frame deletion/insertion (6%)^9^. It is typically believed that up to 5% of individuals with a clinical diagnosis of VHL have negative genetic test results from germline DNA analysis^6–8^. However, a national study in Denmark found no disease-causing *VHL* variant in 21% (15/71) assumed VHL patients that underwent genetic testing, which included patients with a hemangioblastoma of the CNS^10^. This may be due to postzygotic mosaicism, phenotypic coincidence, methylation, alteration in another gene that results in a phenotype similar to VHL, or a cryptic variant in *VHL* not detected by conventional methods^4,11,12^.

Only a few case studies^13–15^ have reported VHL mosaicism; the rate of mosaicism in VHL is largely unknown and may be under-reported. Several studies have attempted to quantify the rate of mosaicism within VHL, the most notable being Sgambati et al (2000)^16^. This study found mosaic variants in ~5% (2/42) of patients with a clinical diagnosis of VHL, but without a family history. A recent study used Next Generation Sequencing (NGS) in combination with single mutation-specific PCR methods in a cohort of gene-negative patients with either clinical VHL or suspected VHL^17^. This combination method was able to detect mosaic variants in 8.5% (4/47) of their cohort^17^. Another paper by the same group performed deep sequencing (>1000×) on eight patients suggestive of VHL, with two patients fulfilling clinical VHL criteria. A pathogenic *VHL* variant was detected at 1.7% and 5.7% variant allele fractions (VAFs) in the two cases with clinical VHL^18^. Of note, tumor testing was performed in only one study where half the patients (2/4) with mosaic variants had tumor tissue analyzed^17^. All studies defined mosaicism in terms of a variant with a low VAF, with no specific cutoff provided.

Advances in NGS allow for high throughput and more sensitive detection of variants at lower VAFs and more cases of mosaic VHL may emerge^19^. To further ascertain and counsel these cases, tumor analysis of the *VHL* gene and tumor immunohistochemistry will become increasingly important. Here, we present a case of a suspected mosaic individual who fulfilled clinical criteria for VHL but initially received negative germline *VHL* results through multigene panel testing on DNA extracted from peripheral blood leukocytes. We describe further evaluation with tumor tissue analysis as a complementary tool in the workup of such variant elusive VHL cases.

## Results

### Patient characteristics

A 55-year-old male presented in the emergency room with shortness of breath, chest pain, and coughing; a chest X-ray found a perihilar mass on the left lung. A CT scan of the chest confirmed the mass on the left lung (measuring 3.1 × 1.8 cm) and noted a left renal lesion. A CT scan of the abdomen and pelvis revealed a large mass on the left kidney and right adrenal gland. An ultrasound guided biopsy showed that the left kidney mass was a renal cell carcinoma. The patient subsequently underwent a left-sided radical nephrectomy and right-sided adrenalectomy. The pathology results confirmed ccRCC (measuring 12 × 11 × 9.5 cm) and identified a pheochromocytoma (measuring 6.7 × 5.8 × 5 cm). Biochemical evaluation of urine and plasma metanephrines and urine catecholamines were within normal limits. Two pieces of lung tissue orally expelled from the patient further supported metastatic ccRCC. While spinal MRI and chest CT identified bone metastases, there were also two spinal lesions (measuring 2.2 mm and 14 mm) consistent with spinal hemangioblastomas. In addition, histologically confirmed liver metastases from ccRCC were noted. The patient succumbed to his disease 10 months after initial presentation. The patient’s family history was unremarkable for VHL and no other VHL-associated manifestations were found on the patient’s subsequent imaging. A clinical diagnosis of Type 2B VHL was presumed, and blood was sent for germline molecular genetic testing.

### Initial genetic testing results

The patient underwent germline testing on DNA isolated from peripheral blood leukocytes with a panel of genes targeted at hereditary renal cell cancer and a hereditary paraganglioma-pheochromocytoma. The patient tested negative for any pathogenic, likely pathogenic, or uncertain DNA sequence or copy number variants in *FH, FLCN, MET, MITF, MLH1, MSH2, MSH6, PMS2, PTEN, SDHA, SDHB, SDHC, SDHD, TP53, TSC1, TSC2*, *MAX, MEN1, NF1, RET, SDHAF2, TMEM127*, and *VHL*. Variant allele threshold for review on the germline panel was 10% or greater VAF.

As a molecular genetic diagnosis of VHL was not identified, the daughters of the proband underwent germline molecular genetic analysis of *VHL* and results were negative. However, given the family history, both daughters were enrolled in full VHL screening including ophthalmologic assessment, brain, spine, and abdominal MRI. They have no VHL manifestations at the ages of 25 and 30.

### Morphological and immunohistochemical characteristics of pheochromocytoma

**Fig. 1:**
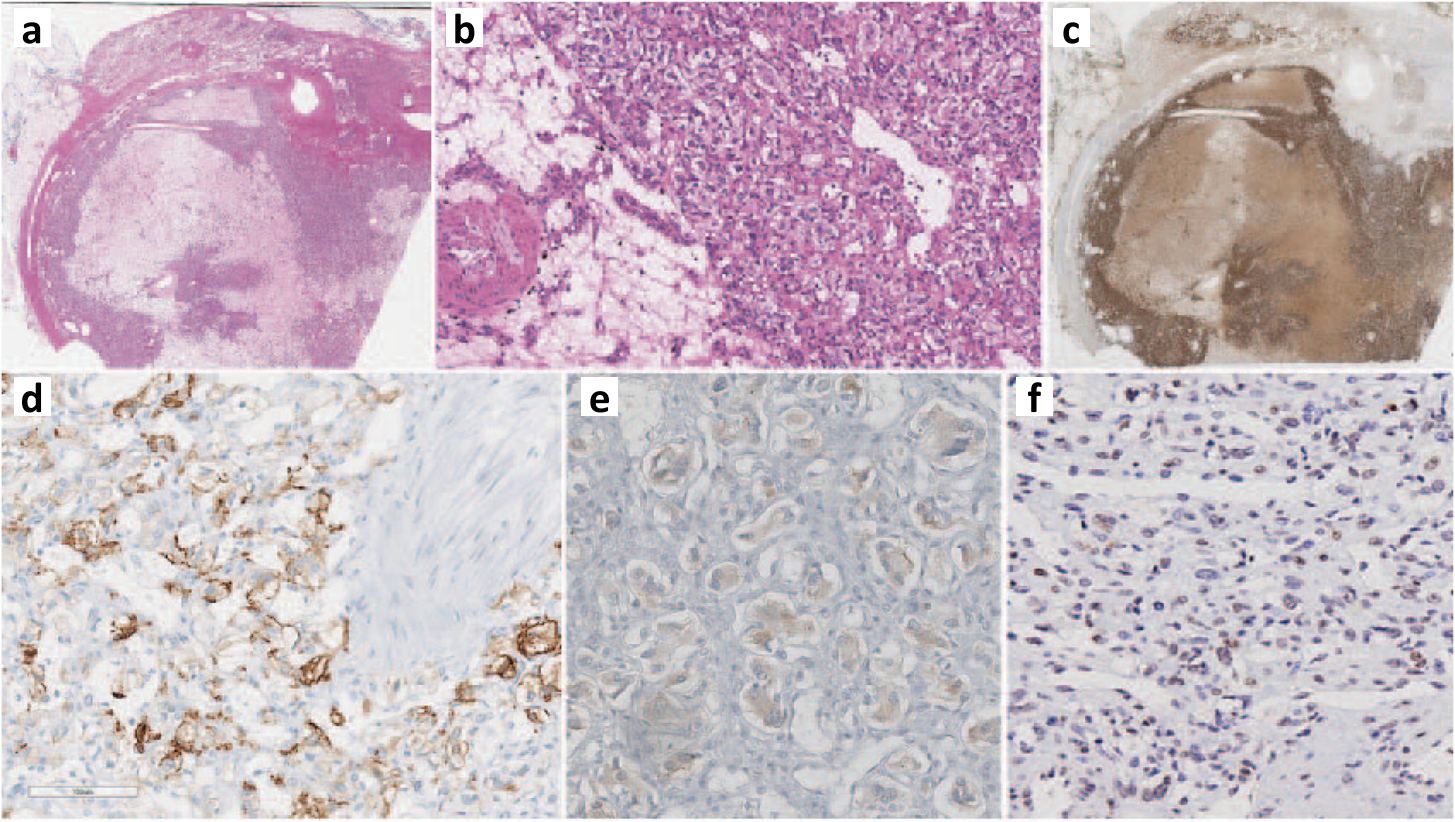
Morphological and immunohistochemical findings of pheochromocytoma. Whole scanned images: The adrenalectomy specimen (hematoxylin and eosin) shows an encapsulated pheochromocytoma with clear cell change, variable fibrohyaline, and myxoid stroma rich in microvasculature (**a, b**). The tumor cells are diffusely positive for tyrosine hydroxylase (the rate limiting enzyme in catecholamine synthesis) (**c**). The tumor is positive for carbonic anhydrase IX (**d**). Alpha-inhibin shows variable weak reactivity in the tumor cells (**e**). pVHL shows variable loss or significantly reduced staining intensity in the tumor cells (**f**).

The adrenal tumor tissue underwent detailed morphological assessment by an endocrine pathologist. The adrenal tumor was an encapsulated pheochromocytoma with variable clear cell change and stromal degeneration with increased microvasculature (Fig. 1a, b). For immunohistochemistry analysis, positivity for tyrosine hydroxylase (Fig. 1c) and GATA3 confirmed the diagnosis of pheochromocytoma. The cytomorphological findings (e.g., clear cell change, encapsulation with stromal changes) were highly suggestive of *VHL-*related pheochromocytoma^20^. While there was no cyclinD1 overexpression, the tumor was positive for carbonic anhydrase IX (CAIX) and alpha-inhibin (variable and weak) (Fig. 1d, 1e). Alpha-inhibin expression^21^, along with CAIX expression^21–23^, was consistent with VHL-disease-related pseudohypoxia, especially in the background of morphological findings. Moreover, variable loss of pVHL also supported an altered pseudohypoxia pathway in this tumor (Fig. 1f).

### Genetic testing of tumor specimens

**Fig. 2:**
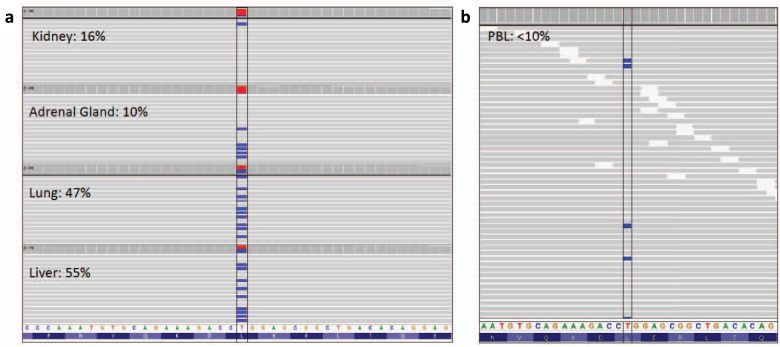
Visualization of *VHL* variant in tumor and germline DNA. Integrative Genomics Viewer display of the c.593 T > C (p.Leu198Pro) variant in tissue from four tumor specimens (**a**) and peripheral blood leukocytes (**b**). The percentage of sequencing reads (gray bars) depicting the variant are beside the biospecimen type. The blue bars depict a cytosine nucleotide instead of a thymine nucleotide at the variant loci. The overall proportion of cytosine:thymine reads is represented by the blue:red bars at the top of each tissue panel. Tumor cellularity was estimated by a pathologist to be 65% in the kidney tissue and 85% in the adrenal gland, lung, and liver tissue.

Paraffin embedded tissue retrieved from the ccRCC, pheochromocytoma, expelled lung tissue (sputum), and liver biopsy were tested on a targeted hereditary cancer panel^24^. A likely pathogenic variant in exon 3 of *VHL* (NM_0000551.3: c.593 T > C; p.Leu198Pro) was detected in all four tissues at varying VAFs (Fig. 2). This missense variant is consistent with Type 2 VHL and has been classified as likely pathogenic in the germline context^25–27^. Although no second hits in *VHL* were detected, copy number calling revealed evidence of 3p loss-of-heterozygosity in all tumor tissues (kidney, adrenal, lung, and liver).

The *VHL* p.Leu198Pro variant has been previously reported in the germline of two families^25–27^ and tumor of a patient with sporadic pheochromocytoma^28^. In the first family, three members carried the variant and all three had bilateral pheochromocytomas and one had an additional sympathetic paraganglioma^25,27^. A protein prediction research software (symphony) predicted that this missense variant affects a VHL surface protein residue^25,29^. In the second family, one member presented with an early onset pancreatic neuroendocrine tumor, bilateral pheochromocytomas, and an optic nerve hemangioblastoma^26^.

### Reanalysis of germline testing results

Re-examination of the NGS data from the proband’s initial hereditary cancer testing identified the T allele at c.593 in the *VHL* gene in ~6% of the sequencing reads (77 reads of the total 1298 at this base pair position). This was below the typical reportable threshold for NGS multigene panel testing of 10%, and therefore was not initially reported in clinical genetic testing.

## Discussion

Approximately 95–100% of individuals with clinical VHL receive a positive result when they undergo standard genetic testing on DNA extracted from blood or saliva. This case report explores the rare event of an individual with typical VHL manifestations testing negative for a *VHL* variant on germline DNA extracted from peripheral blood leukocytes, with subsequent sequencing of four postmortem tumor tissue specimens revealing a *VHL* pathogenic variant in all four tissues. Tumor analysis revealed protein markers consistent with *VHL*-driven tumorigenesis and pVHL. Reanalysis of germline genetic testing results on the blood sample detected the variant at 6% VAF, confirming the strong likelihood of somatic mosaicism in this case.

This case illustrates several factors to consider for conditions where somatic mosaicism is suspected based on the proband and family history. Clinical genetic testing results are typically anticipated to be categorical (wild-type, heterozygous, and homozygous), as this most accurately represents the inheritance of Mendelian disease-causing variants. The targeted panel performed on DNA isolated from the patient’s peripheral blood leukocytes was validated to detect variants above a 10% VAF, as this was the expected lower VAF range for a germline heterozygous variant on this panel. Variants present at reduced VAFs may not be reported in all clinical labs. In addition, the lower limit threshold for reporting variants is not a standard value for NGS panels whose intended use is to detect inherited germline variants expected to be heterozygous or homozygous. Communication with the testing laboratory, including clinical suspicions for a specific genetic diagnosis, can be helpful in triggering re-examination of low-level calls or raw data for potentially mosaic results. In addition, negative genetic testing should not be considered as a definitive conclusion and further investigations, including of other tissue types, may be required to arrive at a diagnostic molecular finding. Our VAF of the various affected tissues ranged from as low as 10% to as high as 55% despite adequate tumor cellularity. This variability may be due to tumor biology such as inflammatory cells causing normal tissue contamination and other genomic events and is a reported limitation of VAF from tumor sequencing^30^.

Mosaicism results from the acquisition of a somatic mutation during embryogenesis, leading to variable mutant cell frequency throughout the body. While previously reported that individuals with mosaic VHL are asymptomatic or present with a mild phenotype^13–15^, Coppin et al. (2014) have reported a severe phenotype in a mosaic VHL patient^18^. Here we presented an individual with three VHL-associated manifestations, which provides further evidence that mosaicism in VHL can present with a severe phenotype. Cases of mosaicism in similar Mendelian conditions have been described previously with phenotypes less severe than might be expected from these autosomal dominant conditions; however, given the incomplete penetrance and variable expressivity of these conditions themselves, it is difficult to make specific phenotypic predictions^31,32^. A systematic review of 111 articles reporting on 320 mosaic NF1 individuals found that mosaic NF1 patients present with a milder phenotype and have fewer complications and manifestations^31^. A study on 39 Tuberous Sclerosis Complex patients with mosaicism (<10% VAF) also found that mosaic patients presented with a milder phenotype^32^. Further examination of suspected VHL mosaicism is needed to discern the relationship between phenotype severity and mosaicism in this hereditary cancer syndrome.

Notably, the two offspring of this proband had negative germline testing, which included analysis of *VHL* prior to the identification of this mosaic finding. The eventual identification of a molecular cause for their father’s phenotype may have clinical implications for these two daughters. However, given the findings from this report, the risk of VHL disease to the daughters may be significantly decreased and may play a role in counseling them to continue to undergo intensive VHL surveillance. The current gold-standard for mosaic testing involves analysis of tissue from all three germ layers and may not be possible in many clinical scenarios, including in rapidly deteriorating patients. This case report shows the reality of mosaic testing and proves the value in completing tissue testing on available tissue as the result provides valuable information that can be used to guide the surveillance and treatment of family members.

## Methods

### Ethics approval and clinical trial registration

Written informed consent for this study was obtained from the family of the proband following a clinical research protocol approved by the Research Ethics Board at University Health Network (#16-5831). This research study is registered at clinicaltrials.gov (clinical trials registration number: NCT03857594).

### Genetic analysis

Genomic DNA extracted from peripheral blood leukocytes, and quantified using a spectrophotometer, underwent two targeted NGS panels at a commercial laboratory (Ambry Genetics Corporation, Aliso Viejo, California) for clinical testing. Ambry Genetics is a CLIA-certified, CAP accredited laboratory, which performs clinical genetic testing. The requested assays included NGS of all coding base pairs and 5 base pairs of intronic region flanking the exons of 12 genes associated with hereditary renal cell cancer and hereditary paraganglioma-pheochromocytoma predisposition. The genes and reference sequences used for analysis are: FH- NM_000143, MAX- NM_002382, MEN1-NM_130799.2, NF1- NM_001042492, RET- NM_020975, SDHA- NM_004168, SDHAF2-NM_017841, SDHB- NM_003000, SDHC- NM_003001, SDHD- NM_003002, TMEM127- NM_017849, and VHL- NM_000551. Sequence enrichment of the targeted coding exons and adjacent intronic nucleotides was carried out by a bait-capture methodology using long biotinylated oligonucleotide probes followed by polymerase chain reaction and NGS. Sequence reads were aligned to the reference human genome (GRCh37) using NovoAlign (version 3.02.07; Novocraft Technologies, Selangor, Malaysia) and variant calls generated using the Genome Analysis Toolkit (version 3.2.2; Broad Institute, Cambridge, MA). Suspect variant calls, other than those classified as “likely benign” or “benign”, were verified by Sanger sequencing. A minimum coverage of 25×, Q score of 30 and VAF of >10% were required for candidate variants to pass quality control metrics for reporting. Any regions with an insufficient depth of coverage for heterozygous variant calling were analyzed by Sanger sequencing. The sequence of copy number variants which did not meet internal quality control metrics for confidence were confirmed by a secondary methodology, such as chromosomal microarray or Sanger sequencing, prior to reporting.

Paraffin embedded clinical blocks were retrieved postmortem and DNA from four separate tissues (ccRCC, pheochromocytoma, expelled lung tissue, and liver biopsy). The tissue underwent pathology review by experienced clinical pathologists who determined histology and tumor cellularity. DNA was extracted from the four tissues and used in 59-gene NGS panel (Clinical Genome Diagnostics Laboratory, University Health Network, Toronto, Ontario) for detection of pathogenic variants associated with hereditary cancer syndromes, using methods previously described for analysis of tumor DNA from FFPE material^24^. For variant analysis, a custom bioinformatic pipeline was used with targeted sequence reads aligned to the reference human genome (hg19) using Burrows-Wheeler Aligner (version 0.7.12)^33^. Duplicate reads were marked (Picard Mark Duplicates; version 1.130) and the Genome Analysis Toolkit (version 3.3.0; Broad Institute, Cambridge, MA) best practices followed Base Quality Score Recalibration. Somatic single nucleotide variants and small insertion/deletions were detected by Varscan2 (version 2.3.8) and copy number status was determined by CNVkit (version 0.7.11)^34,35^. Loss-of-heterozygosity was detected using pureCN (version 1.12.0) in R (version 3.5.0), with input data from CNVkit and MuTect (version 1.1.5)^36,37^. All variants were filtered using Alissa software (Agilent, Santa Clara, CA) to detect variants with a VAF > 10% and to remove benign variants. All variants were manually reviewed in IGV (version 2.3)^38^. All genomic regions achieved at least 25× coverage.

### Immunohistochemistry

Immunohistochemical staining for GATA3 (Biocare Medical, Pacjeco, CA; catalog number: BC-CM405bl dilution: 1:100), tyrosine hydroxylase (Abcam, Cambridge, UK; catalog number: ab75875-2; dilution: 1:500), cyclinD1 (Cell Marque, Rocklin, CA; catalog number: CMQ-241R16; dilution: 1:250), alpha-inhibin (Cedarlane, Burlington, ON; catalog number: MCA951S; dilution: 1:100) and CAIX (Leica, Wetzlar, Germany; catalog number: CAIX-L-CE; dilution: 1:100) was performed in our clinical diagnostic immunohistochemistry laboratory using appropriate positive and negative controls. pVHL immunohistochemistry was performed in an institutional research laboratory according to the manufacturer’s instructions (OriGene, Rockville, MD; catalog number: TA506222; dilution 1:500), along with appropriate positive and negative controls.

### Reporting summary

Further information on research design is available in the Nature Research Reporting Summary linked to this article.

## Supplementary information

Reporting Summary

## Data Availability

Tumor bam files from the four tissues that underwent targeted sequencing have been deposited in the European Genome-phenome Archive repository under accession code EGAS00001005895. The germline data derived for this study are not publicly available as they contain information that may compromise the research participant’s privacy but may be accessed by qualified researchers through R.H.K. (raymond.kim@uhn.ca).

## Code availability

No custom code was used to analyze the data and all tools used within the customized bioinformatics pipeline are included in the Methods section, with the version used.
