# Supplementary material for: VHL mosaicism: the added value of multi-tissue analysis
Source: NPJ Genom Med. 2022 Mar 18;7:21. doi: 10.1038/s41525-022-00291-3 (PMC8933488; doi:10.1038/s41525-022-00291-3)
Supplement: Supplementary file 1 — Reporting Summary [file 41525_2022_291_MOESM1_ESM.pdf]

nature portfolio

Corresponding author(s): Raymond Kim
Last updated by author(s): Sep 6, 2021

Reporting Summary

Nature Portfolio wishes to improve the reproducibility of the work that we publish. This form provides structure for consistency and transparency in reporting. For further information on Nature Portfolio policies, see our Editorial Policies and the Editorial Policy Checklist.

Statistics

For all statistical analyses, confirm that the following items are present in the figure legend, table legend, main text, or Methods section.

n/a	Confirmed	
☒	☐	The exact sample size (n) for each experimental group/condition, given as a discrete number and unit of measurement
☒	☐	A statement on whether measurements were taken from distinct samples or whether the same sample was measured repeatedly
☒	☐	The statistical test(s) used AND whether they are one- or two-sided<br>*Only common tests should be described solely by name; describe more complex techniques in the Methods section.*
☒	☐	A description of all covariates tested
☒	☐	A description of any assumptions or corrections, such as tests of normality and adjustment for multiple comparisons
☒	☐	A full description of the statistical parameters including central tendency (e.g. means) or other basic estimates (e.g. regression coefficient) AND variation (e.g. standard deviation) or associated estimates of uncertainty (e.g. confidence intervals)
☒	☐	For null hypothesis testing, the test statistic (e.g. F, t, r) with confidence intervals, effect sizes, degrees of freedom and P value noted<br>*Give P values as exact values whenever suitable.*
☒	☐	For Bayesian analysis, information on the choice of priors and Markov chain Monte Carlo settings
☒	☐	For hierarchical and complex designs, identification of the appropriate level for tests and full reporting of outcomes
☒	☐	Estimates of effect sizes (e.g. Cohen's d, Pearson's r), indicating how they were calculated

Our web collection on statistics for biologists contains articles on many of the points above.

Software and code

Policy information about availability of computer code

Data collection	No software was used for data collection.
Data analysis	Genomic DNA extracted from peripheral blood leukocytes, and quantified using a spectrophotometer, underwent two targeted NGS panels at a commercial laboratory (Ambry Genetics Corporation, Aliso Viejo, California). Sequence reads were aligned to the reference human genome (GRCh37) using NovoAlign (version 3.02.07; Novocraft Technologies, Selangor, Malaysia) and variant calls generated using the Genome Analysis Toolkit (version 3.2.2; Broad Institute, Cambridge, MA). Suspect variant calls, other than those classified as "likely benign" or "benign", were verified by Sanger sequencing. A minimum coverage of 25x, Q score of 30 and VAF of >10% were required for candidate variants to pass quality control metrics for reporting.<br><br>Paraffin embedded clinical blocks from four tumor tissues underwent a 59-gene NGS panel. For variant analysis, a custom bioinformatic pipeline was used with targeted sequence reads aligned to the reference human genome (hg19) using Burrows-Wheeler Aligner (version 0.7.12). Duplicate reads were marked (Picard Mark Duplicates) and the Genome Analysis Toolkit (version 3.3.0; Broad Institute, Cambridge, MA) best practices followed Base Quality Score Recalibration. Somatic single nucleotide variants and small insertion/deletions were detected by Varscan2 (version 2.3.8) and copy number status was determined by CNVkit (version 0.7.11). Loss-of-heterozygosity was detected using pureCN (version 3.5.0 of R). All variants were filtered using Alissa software (Agilent, Santa Clara, CA) to detect variants with a VAF >10% and to remove benign variants. All variants were manually reviewed in IGV (version 2.3).

For manuscripts utilizing custom algorithms or software that are central to the research but not yet described in published literature, software must be made available to editors and reviewers. We strongly encourage code deposition in a community repository (e.g. GitHub). See the Nature Portfolio guidelines for submitting code & software for further information.

Data

Policy information about availability of data

All manuscripts must include a data availability statement. This statement should provide the following information, where applicable:

- Accession codes, unique identifiers, or web links for publicly available datasets
- A description of any restrictions on data availability
- For clinical datasets or third party data, please ensure that the statement adheres to our policy

Data from this study are available upon request from the corresponding author (R.H.K.). The data derived for this study are not publicly available as they contain information that may compromise the research participant's privacy.

Field-specific reporting

Please select the one below that is the best fit for your research. If you are not sure, read the appropriate sections before making your selection.

☒ Life sciences ☐ Behavioural & social sciences ☐ Ecological, evolutionary & environmental sciences

For a reference copy of the document with all sections, see nature.com/documents/nr-reporting-summary-flat.pdf

Life sciences study design

All studies must disclose on these points even when the disclosure is negative.

Sample size	One.
Data exclusions	No data was excluded.
Replication	None.
Randomization	None.
Blinding	None.

Reporting for specific materials, systems and methods

We require information from authors about some types of materials, experimental systems and methods used in many studies. Here, indicate whether each material, system or method listed is relevant to your study. If you are not sure if a list item applies to your research, read the appropriate section before selecting a response.

Materials & experimental systems

n/a	Involved in the study	
☐	☒	Antibodies
☒	☐	Eukaryotic cell lines
☒	☐	Palaeontology and archaeology
☒	☐	Animals and other organisms
☐	☒	Human research participants
☐	☒	Clinical data
☒	☐	Dual use research of concern

Methods

n/a	Involved in the study	
☒	☐	ChIP-seq
☒	☐	Flow cytometry
☒	☐	MRI-based neuroimaging

Antibodies

Antibodies used	Clinical immunohistochemical staining performed for: GATA3, tyrosine hydroxylase, cyclinD1, alpha-inhibin and CAIX. pVHL immunohistochemistry was performed in an institutional research lab.<br>GATA-3: Biocare Medical; BC-CM405b; 1:100<br>CyclinD1: Cell Marque; CMQ-241R16; 1:250<br>Alpha-inhibin: Cedarlane; MCA951S; 1:100<br>CAIX: Leica; CAIX-L-CE; 1 :100<br>Tyrosine Hydroxylase: Abcam; ab75875-2; 1:500<br><br>The pVHL antibody was purchased from OriGene, catalog number: TA506222, dilution 1:500.
Validation	Unknown.

Human research participants

Policy information about studies involving human research participants

Population characteristics	Case report of a 55-year-old male with clinical VHL disease.
Recruitment	Participant's family provided consent for us to complete genomic analyses and clinical data collection on deceased proband.
Ethics oversight	University Health Network

Note that full information on the approval of the study protocol must also be provided in the manuscript.

Clinical data

Policy information about clinical studies

All manuscripts should comply with the ICMJE guidelines for publication of clinical research and a completed CONSORT checklist must be included with all submissions.

Clinical trial registration	NCT03857594
Study protocol	Study protocol is available upon request.
Data collection	Participant was deceased at time of enrolment into the study- data was collected from time presented in the emergency room to death.
Outcomes	N/A
